# Dietary Copper Plays an Important Role in Maintaining Intestinal Barrier Integrity During Alcohol-Induced Liver Disease Through Regulation of the Intestinal HIF-1α Signaling Pathway and Oxidative Stress

**DOI:** 10.3389/fphys.2020.00369

**Published:** 2020-05-08

**Authors:** Hongwei Lin, Dazhi Chen, Qianjing Du, Tongtong Pan, Hanxiao Tu, Yuedong Xu, Teng Teng, Jingjing Tu, Ji Li, Zhuo Lin, Xiaodong Wang, Lanman Xu, Yong-Ping Chen

**Affiliations:** ^1^Department of Infectious Diseases, The First Affiliated Hospital of Wenzhou Medical University, Zhejiang Provincial Key Laboratory for Accurate Diagnosis and Treatment of Chronic Liver Diseases, Hepatology Institute of Wenzhou Medical University, Wenzhou, China; ^2^Department of Gastroenterology, Peking University First Hospital, Beijing, China

**Keywords:** copper, HIF-1α, alcoholic liver injury, intestinal barrier, Caco-2

## Abstract

Impaired intestinal barrier function and oxidative stress injury play critical roles in the pathogenesis of alcoholic liver disease (ALD), and recent investigations have revealed a role for dietary copper in the liver and intestinal barrier function. Therefore, the current study investigates the mechanisms and role of dietary copper in alcohol induced liver diseases. C57BL/6 mice were used to create an alcoholic liver disease model with a Lieber-DeCarli diet containing 5% alcohol and were fed with different concentrations of dietary copper of adequate (6 ppm, CuA), marginal (1.5 ppm, CuM), or supplemental (20 ppm, CuS) amounts. Caco-2 cells were also exposed to ethanol and different concentrations of copper. Damages of the liver and intestine were evaluated by transaminases, histology staining, and protein and mRNA level, as well as cell proliferation, oxidative stress, and mitochondrial membrane potential. In animal experiments, the results indicate that an alcohol diet causes liver injury and disruption of intestinal barrier function as well as decreasing the expression of genes such as HIF-1α, occludin, SOD1, and GPX1. Supplemental dietary copper can revert these changes except for SOD1, but marginal dietary copper can worsen these changes. The *in vitro* cell experiments showed that proper copper supplementation can promote cell growth and reduce reactive oxygen species (ROS) production. In conclusion, supplemental dietary copper has beneficial effects on alcohol-induced intestine and liver injury, and marginal dietary copper shows detrimental effects on these parameters.

## Introduction

Alcoholic liver disease (ALD) arises due to excess use of alcohol for a long period of time and ranges from hepatic steatosis to steatohepatitis, cirrhosis, and even hepatocellular carcinoma (Seitz et al., 2018). ALD is a leading cause of morbidity and mortality worldwide, but its targeted therapy is limited (Bajaj, 2019; Ge et al., 2019). Therefore, research on mechanisms and treatment methods is urgently needed. Although the pathogenesis of ALD may involve many aspects (Rao, 2009; Gao and Bataller, 2011; Orman et al., 2013; Chen et al., 2016), recent studies have suggested that gut barrier dysfunction induced by alcohol, such as increased intestinal permeability, bacterial translocation, and release of bacteria-derived endotoxin into circulation, may play an important role in ALD development.

The barrier function of intestinal epithelial cells is regulated by a variety of factors, including oxygen (Rath et al., 2018). Moreover, alcohol-induced oxidative stress and inflammatory reactions can aggravate tissue hypoxia (Louvet and Mathurin, 2015). Since hypoxia-inducible factor-1α (HIF-1α) is a regulatory subunit of the hypoxia-inducible factor (HIF), which regulates a wide range of genes involved in cellular responses to hypoxia and other tissue environmental cues (Prabhakar and Semenza, 2012), HIF-1α should play an important role in maintaining barrier functions of intestinal epithelial cells. In addition, HIF-1α was significantly increased in hepatic fibrotic tissues and activated hepatic stellate cells (HSCs) (Moon et al., 2009), which are the consequences of alcoholic diseases. However, the role of HIF-1α in alcoholic diseases still remains to be investigated.

An adequate supply of copper is essential for healthy life, as chronic copper deficiency can elicit anemia, leucopenia, myelopathy, or skin abnormalities (Altarelli et al., 2019). Moreover, copper deficiency in rodent shows an adverse effect on lipid metabolism (Al-Othman et al., 1992, 1993). Furthermore, several investigations revealed that copper might be involved in the regulation of gut microbiota and gut barrier function (Song et al., 2018). For example, copper has been used as an antimicrobial agent throughout the ages (Hodgkinson and Petris, 2012), and the response to copper stress varies greatly among different bacterial species (Pontel and Soncini, 2009; Solioz et al., 2010). However, there are no copper-related drugs for the clinical treatment of alcoholic liver. In addition, low copper availability was observed in NAFLD patients, and a copper-deficient diet induces fatty liver in rodents (Aigner et al., 2008, 2010). However, whether and how dietary copper contributes to the development of ALD through altering gut barrier function and oxidative stress remain unknown. Therefore, in this research, we employed mice and Caco-2 cells to investigate the role of dietary copper in the development of alcoholic hepatic steatosis and intestinal epithelial functions.

## Materials and Methods

### Animal Experiments

**FIGURE 1:**
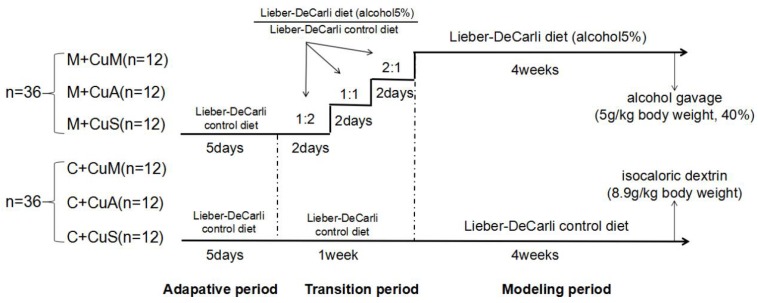
Animal feeding schedule. M + CuM: model group + marginal Cu level, M + CuA: model group + adequate Cu level, M + CuS: model group + supplemental Cu level, C + CuM: control group + marginal Cu level, C + CuA: control group + adequate Cu level, C + CuS: control group + supplemental Cu level.

As shown in Figure 1, seventy-two C57BL/6 wild type (WT) mice (6 weeks of age, male) from the Shanghai Laboratory Animal Center (Shanghai, China) were fed (ad lib.) with the Lieber-DeCarli control diet (Trophic Animal Feed High-tech Co., Jiangsu, China) with a defined copper content in the form of cupric carbonate (Bertola et al., 2013). In order to adapt the animals from solid feed to liquid feed, the mice received 1.5, 6.0, or 20 ppm of copper as marginal, adequate, or supplemental doses, respectively, for 5 days. They were then divided into two groups: a control group (C) and an alcoholic steatosis model group (M). For the purpose of reducing the impact of alcohol on animal function and decreasing mortality, mice were fed a mixed diet of which the ratio (Lieber-DeCarli alcohol diet: Lieber-DeCarli control diet) changed from 2:1 to 1:1 to 1:2 at days 2, 4, and 6, respectively, for 1 week in the model group and were then fed with the Lieber-DeCarli alcohol diet to general steatosis model. In the control group, mice were fed with the Lieber-DeCarli control diet with the same copper content as mice in the model group for 6 weeks. On the 28th day, a bolus of ethanol (5 g/kg body weight, 40%) or isocaloric dextrin (8.9 g/kg body weight) was gavaged in the model and control groups, respectively. After 10 h of gavage, all mice were euthanized to collect blood, liver, and intestinal tissue. Mice were treated according to the protocols reviewed and approved by the Institutional Ethics Committee of Wenzhou Medical University, which were in accordance with the Guiding Principles for the Care and Use of Laboratory Animals in China.

### Measurement of Serum Copper, ALT, and AST Levels

The collected blood was centrifuged at 1500 rpm for 10 min to obtain serum. The levels of serum copper, ALT, and AST were measured by using an automated biochemical analyzer (Abbott Laboratories, Chicago, IL, United States) in the clinical biochemical laboratory of the First Affiliated Hospital of Wenzhou Medical University.

### Liver and Small Intestine Histopathology

Harvested liver and upper small intestine samples were fixed in 4% paraformaldehyde and embedded in paraffin, then cut into 4-μm sections and processed for staining with hematoxylin and eosin (H&E). Moreover, sections of the liver were quickly frozen and then cut into 4-μm sections and stained with Oil Red O. Both were analyzed by light microscopy.

### Real-Time Reverse Transcriptase-Polymerase Chain Reaction (RT-PCR) Assay

Total RNA in tissues was separately extracted according to the instructions of the RNA extraction kit (Aidlab Biotechnologies Co., Beijing, China), and 1 μg total RNA was reverse-transcribed into cDNA using the PrimeScriptTM RTreagent Kit (Perfect Real Time; Aidlab Biotechnologies Co., Beijing, China). Genes related to HIF-1α and small intestine functions (Occludin, SOD1, and GPX1) were examined. The relevant cDNA was then amplified using the SYBR PremixExTaq II (TOYOBO, Osaka, Japan) with specific primers as listed in Table 1. RT-PCR was performed by a 7500 Real-Time PCR System (Applied Biosystems, Life Technologies, Waltham, MA, United States). Each mRNA expression was calculated based on glyceraldehyde-3-phosphate dehydrogenation (GAPDH) as a reference gene. The final mRNA expression was analyzed by the 2^−ΔΔ*Ct*^ method.

**TABLE 1:** Primer sequences used in RT-PCR.

Name	Primer sequences	Tm	Length of product
HIF-1α	Forward primer: ATGCTAAATCGGAGGGTA Reverse primer: TCCTGGAAACGAGTGAAA	60	88
Occludin	Forward prime: CCATCTTTCTTCGGGTTT Reverse primer: TGGATCTATGTACGGCTCAC	60	203
SOD1	Forward primer: GGACCTCATTTTAATCCTCACTCTA Reverse primer: CACACGATCTTCAATGGACACA	60	129
GPx1	Forward primer: GAAGGTAAAGAGCGGGTGA Reverse primer: CAGAATGGCAAGAATGAAGAG	60	132
GAPDH	Forward primer: AAGAAGGTGGTGAAGCAGG Reverse primer: GAAGGTGGAAGAGTGGGAGT	60	111

### Western Blot Analysis

Total protein from tissues was extracted in a mixture of ice-cold RIPA (Beyotime Biotechnology, China) and protease inhibitor. Proteins were separated by 10% SDS-PAGE and electro-transferred to PVDF membrane (Millipore, Burlington, MA, United States). The membranes were blocked for 2 h at room temperature with 5% BSA in TBST solution and then incubated with primary antibodies of anti-HIF-1α (1:1000, Abcam), anti-Occludin (1:1000, Proteintech), anti-SOD1 (1:1000, Proteintech), anti-GPX1 (1:1000, Affity) and anti-βactin (1:1000, Abcam), respectively, at 4°C overnight. After being washed with TBST, the membranes were incubated with the secondary antibody (goat anti-rabbit) (1:5000, Biosharp) for 1 h and were then washed three times with TBST. The bands were then detected and analyzed by Western Bright ECL (Advansta, San Jose, CA, United States) and Image Lab 4.1 software (Bio-Rad).

### Caco-2 Cell Culture

Caco-2 cells were obtained from the Cell Bank of the Chinese Academy of Sciences (Shanghai, China) and cultured in DMEM (Gibco, United States) containing 10% fetal bovine serum (Gibco, United States), penicillin (100 U/ml), and streptomycin (100 U/ml) in a 5% CO_2_ humidified incubator at 37°C. The medium was changed every 2 days, and the growth of the cells was observed with an inverted microscope. The cell monolayer was used after 24 days of culture, at which time the cells have differentiated and matured. The cells were exposed to 100 mmol/L alcohol and Cu (added as CuSO_4_.5H_2_O; Macklin, Shanghai, China) with different concentrations (0, 3.125, 6.25, 12.5, and 25 μM).

### Proliferation Assay

The effect of different concentrations of copper on Caco-2 proliferation was determined using real-time cellular analysis (RTCA). After 24 h stimulation, cells (8 × 10^3^ cells/well) were seeded in several E-Plate 16 dishes (ACEA Biosciences Inc., CA, United States) for proliferation assays. The plates were kept in the cell incubator at 37°C with 5% CO_2_ for 3–4 days. The cell index and growth curves were automatically recorded on the xCELLigence RTCA System (ACEA Biosciences Inc., CA, United States).

### Mitochondrial Membrane Potential Determination

The mitochondrial membrane potential (ΔΨm) was detected according to the manual of the JC-1 Assay Kit (Beyotime, Jiangsu, China). Briefly, the cells were suspended in 1 ml PBS after 48 h of stimulation at approximately 1 × 10^6^ cells/mL and were incubated with 2 μM of JC-1 for 15 min at 37°C. The cells were washed and resuspended in 500 μL PBS and then analyzed on a flow cytometer with 488 nm excitation and emission at 590 nm (red) and 540 nm (green).

### Cellular Total ROS Level Measurement

The relative level of cellular total reactive oxygen species (ROS) level was detected according to the Reactive Oxygen Species Assay Kit (Beyotime, Jiangsu, China). The cells were collected and incubated with DCFH-DA probe solution in a 37°C, 5% CO_2_ incubator for 20 min (final concentration is 10 μM). Next, they were centrifuged at 1000 rpm for 4 min and then washed three times with FBS-free DMEM to remove the residual probe. Finally, cell samples were resuspended in FBS-free DMEM and subjected to flow cytometry analysis. The mean value of fluorescence intensity indicates the relative level of cellular total ROS level.

Both mitochondrial membrane potential and cellular total ROS level were examined by using a BD Accuri C6 (BD Biosciences, Franklin Lakes, NJ, United States) flow cytometer.

### Statistics and Data Analysis

Comparisons between multiple sets of data were performed using one-way analysis of variance (ANOVA), LSD, and Dunnett T3 tests in SPSS 22.0 software. Data are expressed as mean ± SD. *P* < 0.05 was considered statistically significant.

## Results

### Effects of Different Doses of Dietary Copper on Body Weight, Liver Weight, and Liver/Body Weight Ratio in Mice

**FIGURE 2:**
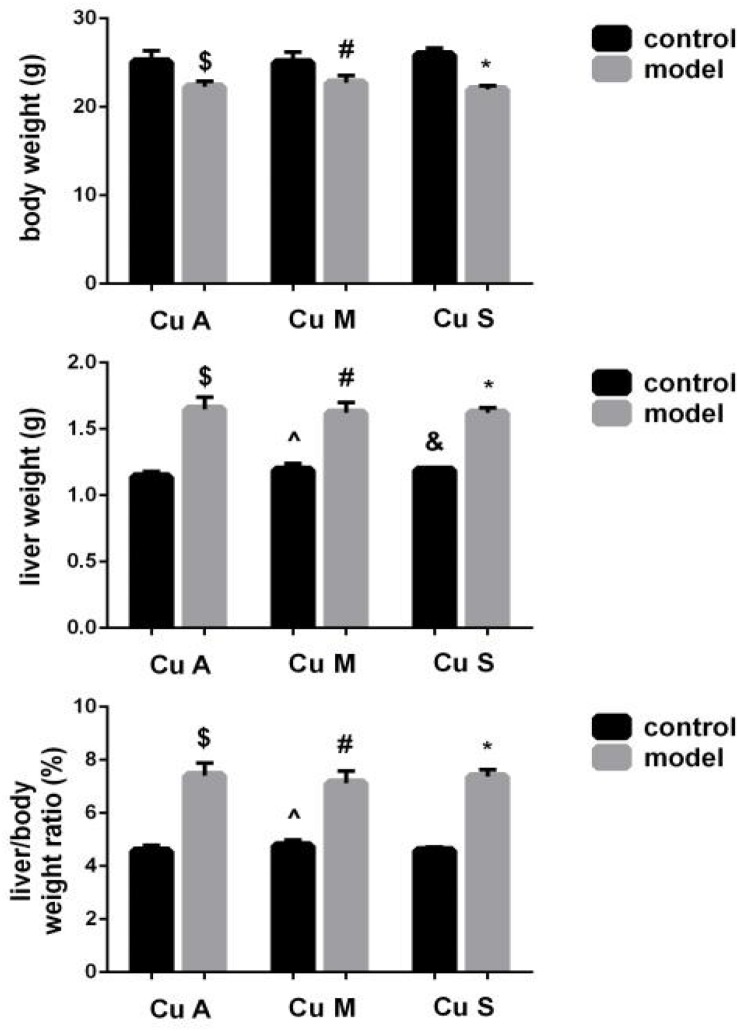
Effects of different doses of dietary copper on body weight, liver weight, and liver/body weight ratio in mice. Comparisons between multiple sets of data were performed using one-way analysis of variance (ANOVA). Data are presented as the mean ± SD (*n* = 12) of three independent experiments. Statistical significance was set at *p* < 0.05. $: M + CuA versus C + Cu A; #: M + CuM versus C + CuM; *: C + CuS versus C + CuS; ^: C + CuM versus C + CuA; &: C + CuS versus C + CuA; + : M + CuM versus M + CuA; ± : M + CuS versus M + CuA.

As shown in Figure 2, alcohol feeding resulted in a significant decrease in body weight but a significant increase in liver weight and liver/body weight ratio. In addition, although the marginal copper diet significantly increased liver weight and the liver/body weight ratio in the control group, this has little practical significance. However, there was no obvious effect of copper on body weight or the liver/body weight ratio in the model group. The results showed that alcohol could damage the liver and cause liver fat deposition but that a beneficial effect of copper supplementation in the diet was not obvious. In the control group, the marginal copper diet was harmful to the liver.

### Serum Copper, ALT, and AST Levels in Response to Different Doses of Dietary Copper in Mice

**FIGURE 3:**
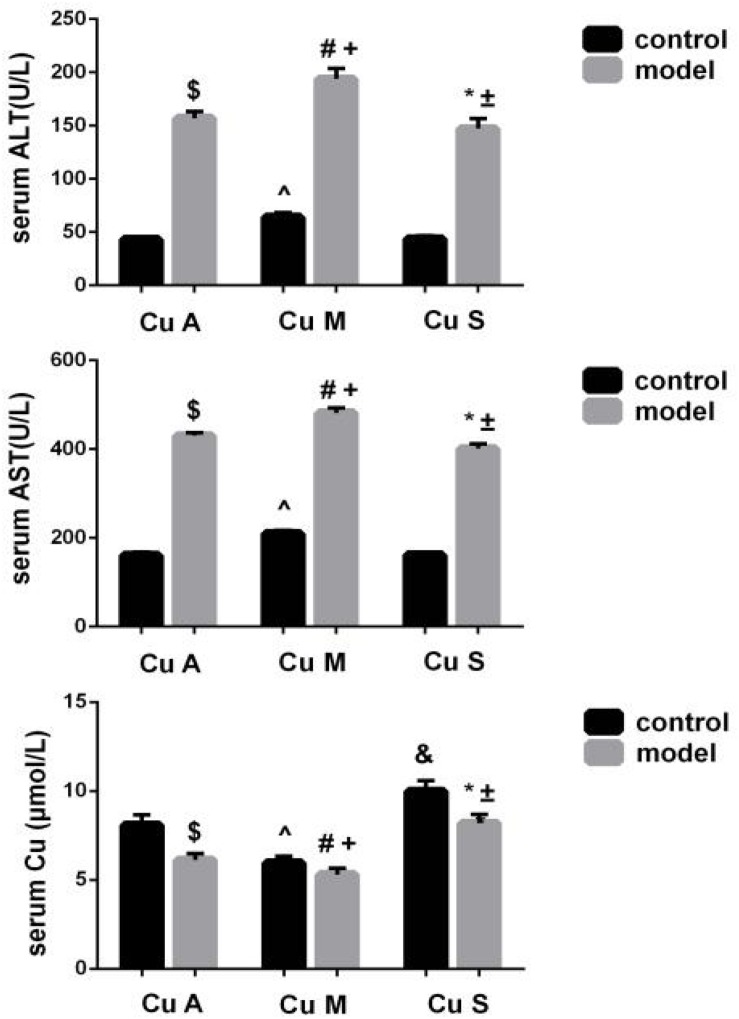
Serum copper, ALT, and AST levels in response to different dietary doses of copper feeding, with or without alcohol. Comparisons between multiple sets of data were performed using one-way analysis of variance (ANOVA). Data are presented as the mean ± SD (*n* = 12) of three independent experiments. Statistical significance was set at *p* < 0.05. $: M + CuA versus C + CuA; #: M + CuM versus C + CuM; *: C + CuS versus C + Cu S; ^: C + CuM versus C + CuA; &: C + CuS versus C + CuA; + : M + CuM versus M + CuA; ± : M + CuS versus M + CuA.

Liver ALT and AST were significantly increased and the serum copper was decreased in the model group (Figure 3). Whether in the control group or the model group, the marginal copper diet can increase ALT and AST and reduce serum copper compared to an adequate copper diet. In contrast, in the model group, copper-supplemented mice had lower ALT and AST values, and serum copper increased. Therefore, alcohol could disrupt the homeostasis of serum copper, damage the liver, and cause elevated aminotransferases. In the model group and the control group, supplemental levels of copper in the diet caused a significant improvement, while marginal copper had a harmful effect.

### Effect of Different Doses of Dietary Copper on Liver Histology in Mice

**FIGURE 4:**
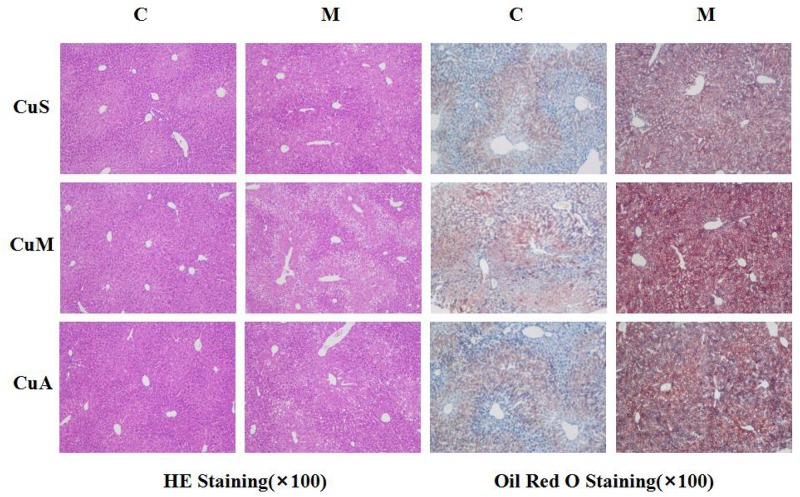
Histology of liver stained with HE and Oil Red O. CuM: marginal Cu level (1.5 ppm); CuA: adequate Cu level (6 ppm); CuS: supplemental Cu level (20 ppm). C: control group; M: model group.

Liver injury was evaluated by liver histology in mice, as shown in Figure 4. Significant liver injury and lipid deposition were observed in the liver of model mice as compared with that in control mice. Moreover, copper supplementation could slightly alleviate liver lipid deposition, while marginal copper exacerbated this change in the control and model groups. The results showed that the damage done by alcohol to the liver was obvious and that copper supplementation in the diet could appropriately alleviate this change, whereas a marginal copper diet exacerbated the situation.

### Effect of Different Doses of Dietary Copper on the Histology and Gene Expression of Small Intestine

**FIGURE 5:**
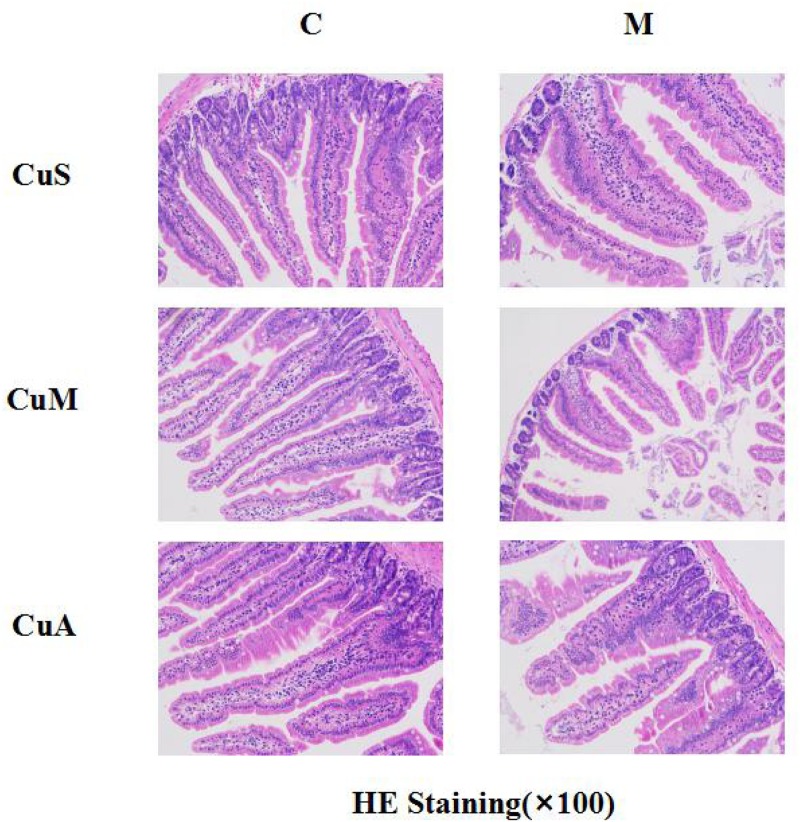
Histology of small intestine stained with HE. CuM: marginal Cu level (1.5 ppm); CuA: adequate Cu level (6 ppm); CuS: supplemental Cu level (20 ppm). C: control group; M: model group.

**FIGURE 6:**
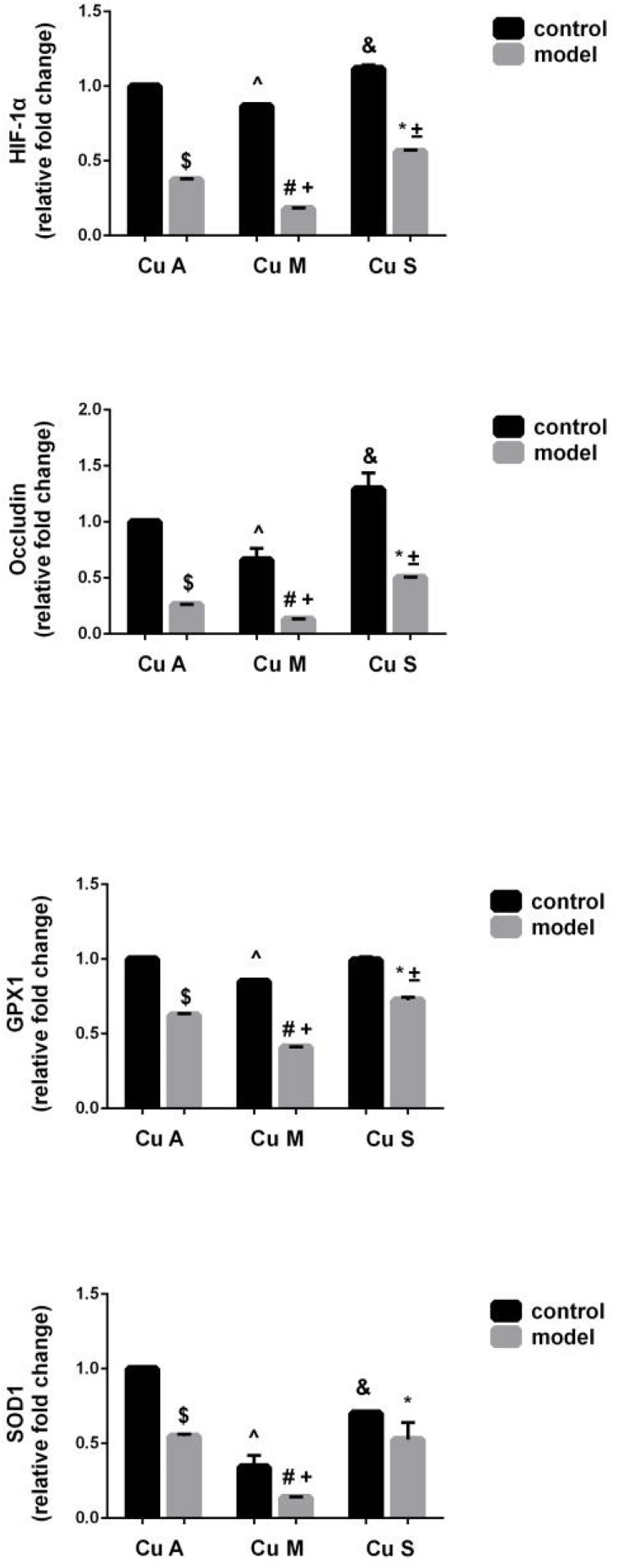
mRNA levels of intestinal HIF-1α, Occludin, GPX1, and SOD1 evaluated by RT-PCR, normalized to the levels of GAPDH. Comparisons between multiple sets of data were performed using one-way analysis of variance (ANOVA). Data are presented as the mean ± SD (*n* = 12) of three independent experiments. Statistical significance was set at *p* < 0.05. $: M + CuA versus C + CuA; #: M + CuM versus C + CuM; *: C + CuS versus C + CuS; ^: C + CuM versus C + CuA; &: C + CuS versus C + CuA; + : M + CuM versus M + CuA; ± : M + CuS versus M + CuA.

**FIGURE 7:**
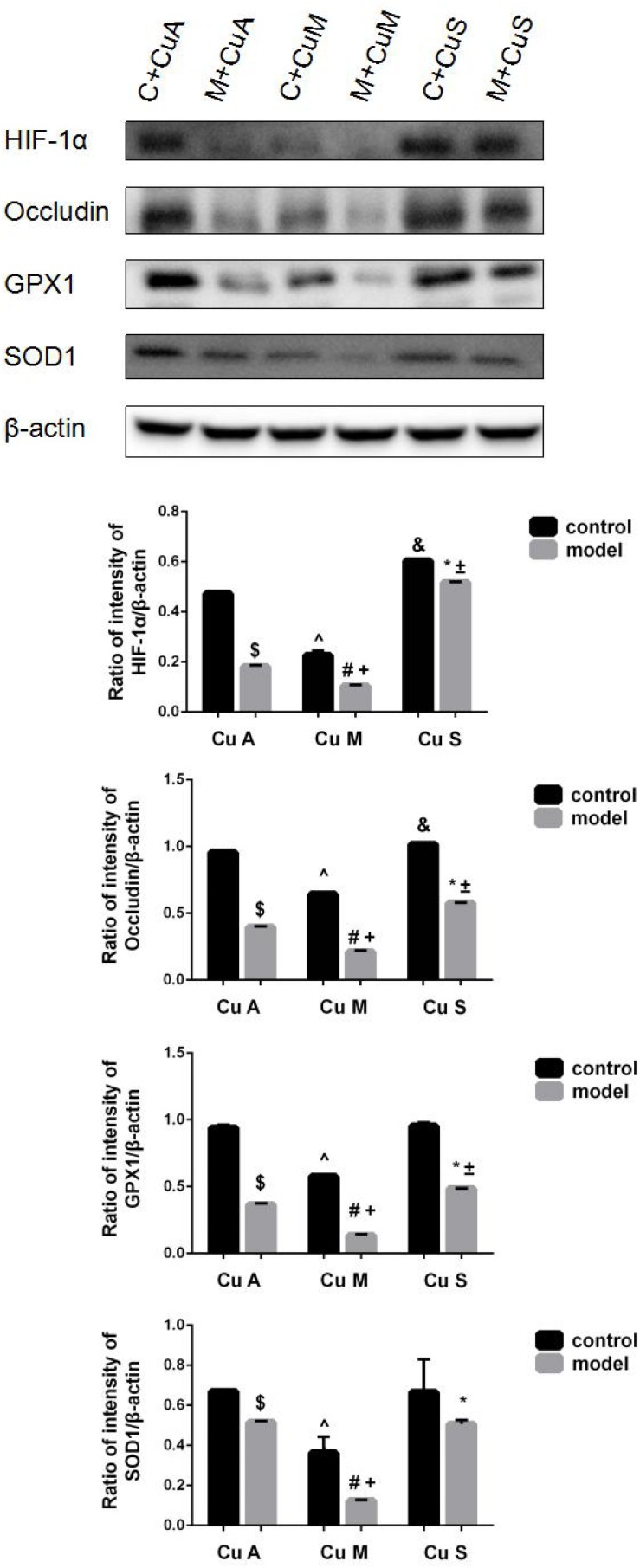
Protein levels of intestinal HIF-1α, Occludin, GPX1, and SOD1 evaluated by WB, normalized to the levels of β-actin. Comparisons between multiple sets of data were performed using one-way analysis of variance (ANOVA). Data are presented as the mean ± SD (*n* = 12) of three independent experiments. Statistical significance was set at *p* < 0.05. $: M + CuA versus C + CuA; #: M + CuM versus C + CuM; *: C + CuS versus C + CuS; ^: C + CuM versus C + CuA; &: C + CuS versus C + CuA; + : M + CuM versus M + CuA; ± : M + CuS versus M + CuA.

The histology of small intestine and gene expression in small intestine are shown in Figures 5–7. As shown in Figure 5, alcohol intake caused the intestinal villi to become thinner, shorter, and more irregular. Moreover, in the model group, the marginal copper diet exacerbated the alcohol-induced intestinal villus destruction, while the supplemental copper diet alleviated it. In the control group, the marginal copper diet also induced changes in intestinal villi, but the supplemental copper diet had no obvious impact on intestinal villi. The genes related to intestinal barrier function and HIF-1α were evaluated, as shown in Figures 6, 7. Alcohol intake resulted in a significant decrease in protein and mRNA levels of occludin, GPX1, and SOD1. The marginal copper diet exacerbated the change, and the supplemental copper diet ameliorated it, except for SOD1 protein expression in the model group. Moreover, alcohol decreased the HIF-1α gene expression in intestine, which suggests a role for HIF-1α in alcohol-induced intestinal injury. The mRNA and protein levels of HIF-1α were lowest in mice fed with the marginal copper diet in both the control and model groups, while the supplemental copper diet appeared to restore the HIF-1α level. The above research results showed that alcohol could damage the intestine by down-regulating the HIF-1α pathway and reducing occludin, SOD1, and GPX1 expression. Copper supplementation in the diet could alleviate these changes, except for that in SOD1, and the marginal copper diet would aggravate all these changes.

### Effect of Different Doses of Dietary Copper on Caco-2 Cells

**FIGURE 8:**
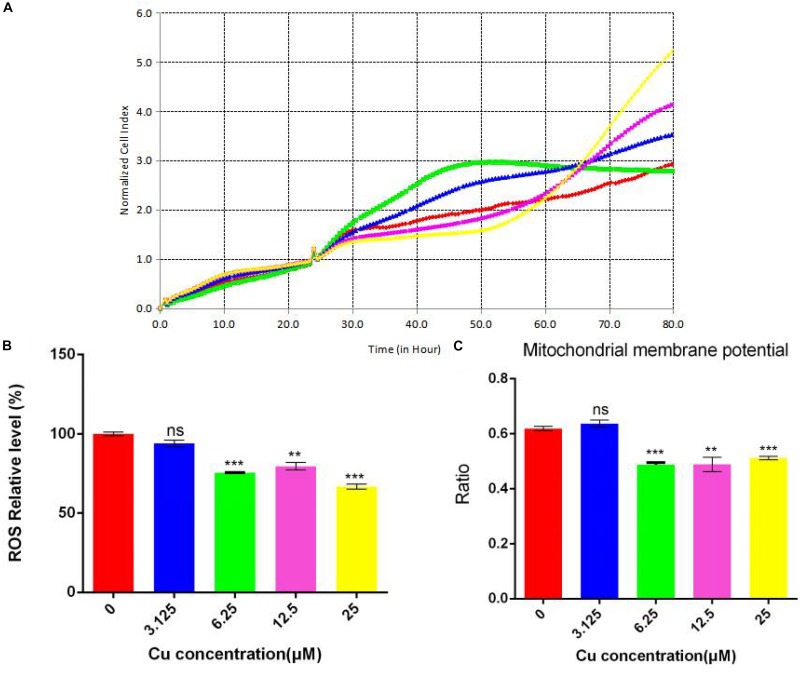
Evaluation of functional mitochondria. **(A)** Proliferation assay real-time cellular analysis (RTCA), **(B)** Cellular total ROS level measurement, **(C)** Mitochondrial membrane potential determination. Comparisons between multiple sets of data were performed using one-way analysis of variance (ANOVA). Data are presented as the mean ± SD (*n* = 12) of three independent experiments. Statistical significance was set at **P < 0.05, ***P < 0.01. ns: not significant.

Since different concentrations of dietary copper can affect liver steatosis and small intestinal barrier function, further investigation of the effects of dietary copper was performed on small intestinal cells (Caco-2 cell line). As shown in Figure 8A, with an increase in the concentration of dietary copper, there was an increase in cell proliferation in an almost dose-dependent manner. Copper also affects ROS production and mitochondrial membrane potential in Caco-2 cells. As shown in Figure 8B, copper decreased ROS production in Caco-2 cells in an almost dose-dependent manner. The same observation was found in mitochondrial membrane potential (Figure 8C). The above results showed that *in vitro* experiments, proper copper supplementation was beneficial for small intestinal cells (Caco-2 cell line) but harmful to mitochondrial membrane potential.

## Discussion

The liver is one of the most important organs of the human body and plays an extremely important role in metabolism (Lackner and Tiniakos, 2019). Since Marshall proposed the concept of the intestinal hepatic axis in 1998, research on the relationship between intestinal and liver diseases has attracted more and more attention (Tripathi et al., 2018). It is currently believed that the intestine and the liver interact with each other, and protection of intestinal mucosal barrier integrity has been a major focus for the development of new treatments for liver diseases (Bird, 2012; Llopis et al., 2016).

The intestines are rich in blood vessels and are therefore susceptible to diseases associated with reduced blood flow and concomitant tissue hypoxia (Taylor and Colgan, 1999; Furuta et al., 2001). Hypoxia-inducible factor 1α (HIF-1α) has been implicated in transcriptional regulation of intestinal barrier integrity and inflammation (Shao et al., 2018). Disruption of epithelial HIF-1α can lead to increased epithelial permeability (Karhausen et al., 2004). Metabolism in the intestine is very active, and thus it is highly vulnerable to oxidative stress (Glover et al., 2016; Rath et al., 2018). Superoxide dismutase 1 (SOD1) and glutathione peroxidase 1 (GPX1) are the most important antioxidant enzymes in cells (Weydert and Cullen, 2010). Tight junction (TJ) is the main means of connection between intestinal epithelial cells and has a major effect on maintaining the polarity of epithelial cells and regulating the permeability of the intestinal barrier (Umeda et al., 2006). Occludin is one of the most important protein molecules in TJ (Du et al., 2010).

In the current study, we found that alcohol intake could induce liver injury and impair gut barrier function. Alcohol caused intestinal villi to become thinner and disordered, decreased the expression of genes such as HIF-1α, occludin, GPX1, and SOD1, and impaired intestinal barrier function. Disorders in intestinal morphology and functions induced by alcohol can be alleviated with supplemental dietary copper and exacerbated with marginal dietary copper. However, supplementation with copper did not produce the expected ameliorative effect on SOD1; it may not be sensitive to increased copper content and may only be more sensitive to copper reduction.

Copper is a necessary mineral element in the diet and has been shown to play roles in antioxidant defense, lipid peroxidation, and mitochondrial function (Al-Othman et al., 1992, 1993). Copper deficiency has been linked to atherogenic dyslipidemia (Aigner et al., 2010). Systemic copper deficiency can also cause mitochondrial dysfunction in mice (Nose et al., 2006), and similar morphological and functional alterations have also been described in human NAFLD (Wei et al., 2008). In the current study, we also found that mice fed with marginal dietary copper also showed damage to the liver and gut without alcohol intake. However, the effect of supplemental dietary copper on control mice was still unclear; it might have a positive effect on the intestine but had no effect on oxidative stress. All of these findings indicated that dietary copper could alter intestinal barrier function through regulation of the intestinal HIF-1α signaling pathway and oxidative stress in mice.

ROS is formed as a natural by-product of the normal metabolism of oxygen and plays an important role in cell signaling and homeostasis. When cell homeostasis is disrupted, ROS levels increase dramatically. This can cause severe damage to the cell structure, which is called oxidative stress. Normal MMP is a prerequisite for maintaining oxidative phosphorylation of mitochondria and producing adenosine triphosphate. The stability of MMP is conducive to maintaining the normal physiological functions of cells. The *in vitro* cell experiments demonstrated an important role of copper in cell proliferation, ROS production, and mitochondrial membrane potential. With an increase in copper concentration, there was an increase in cell proliferation but a decrease in cellular total ROS level. This means that copper could reduce ROS production and promote cell proliferation. Although there is a slight increase in mitochondrial membrane potential at low copper concentration (3.125 μM), a significant decrease in mitochondrial membrane potential was observed with increases in copper concentration. However, the functioning of mitochondria remained intact, which suggested that the membrane potential was more sensitive to changes in the concentration of copper. However, further investigations are needed to understand the relationship between copper and mitochondrial functions.

In summary, our current study provides novel discovery of the pathogenesis of ALD, which indicates that copper homeostasis is critical in maintaining gut barrier integrity and plays an important role in the prevention of ALD. The mechanism by which copper prevents ALD may be related to its regulation of intestinal HIF-1α gene expression and oxidative stress.

## Data Availability Statement

All datasets generated for this study are included in the article/supplementary material.

## Ethics Statement

All mice were treated according to the protocols reviewed and approved by the institutional ethics committee of Wenzhou Medical University.

## Author Contributions

LX and Y-PC designed the protocol. HL and DC performed the experiments and edited the manuscript. QD and TP directed and participated in the sample detection. HT, YX, TT, and JT analyzed the data. HL wrote the manuscript, which was also edited by DC. All authors read and approved the final manuscript.

## Conflict of Interest

The authors declare that the research was conducted in the absence of any commercial or financial relationships that could be construed as a potential conflict of interest.
